# Properties of Hemolysin and Protease Produced by *Aeromonas trota*

**DOI:** 10.1371/journal.pone.0091149

**Published:** 2014-03-14

**Authors:** Eizo Takahashi, Haruka Ozaki, Yoshio Fujii, Hidetomo Kobayashi, Hiroyasu Yamanaka, Sakae Arimoto, Tomoe Negishi, Keinosuke Okamoto

**Affiliations:** 1 Laboratory of Protein Function, Graduate School of Medicine, Dentistry and Pharmaceutical Sciences, Okayama University, Tsushima, Okayama, Japan; 2 Course of Clinical Pharmacy, Yokohama College of Pharmacy, Yokohama, Kanagawa, Japan; 3 Institute of Pharmacognosy, Faculty of Pharmaceutical Sciences, Tokushima Bunri University, Yamashiro, Tokushima, Japan; 4 Laboratory of Molecular Microbiological Science, Faculty of Pharmaceutical Sciences, Hiroshima International University, Kure, Hiroshima, Japan; University of South Florida College of Medicine, United States of America

## Abstract

We examined the properties of exotoxins produced by *Aeromonas trota* (*A. enteropelogenes*), one of the diarrheagenic species of *Aeromonadaceae*. Nine of 19 *A. trota* isolates that grew on solid media containing erythrocytes showed hemolytic activity. However, the hemolytic activities of the culture supernatants of these hemolytic strains of *A. trota* were markedly lower than those of *A. sobria* when cultured in liquid medium, and the amount of hemolysin detected by immunoblotting using antiserum against the hemolysin produced by *A. sobria* was also low. A mouse intestine loop assay using living bacterial cells showed that *A. trota* 701 caused the significant accumulation of fluid, and antiserum against the hemolysin produced suppressed the enterotoxic action of *A. trota* 701. These results indicated that *A. trota* 701 was diarrheagenic and the hemolysin produced was the causative agent of the enterotoxic activity of *A. trota*. The hemolysin in *A. sobria* was previously shown to be secreted in a preform (inactive form) and be activated when the carboxy-terminal domain was cleaved off by proteases in the culture supernatant. Since mature hemolysin was detected in the culture supernatants of *A. trota*, we analyzed the extracellular protease produced by *A. trota*. Fifteen of 19 *A. trota* isolates that grew on solid media containing skim milk showed proteolytic activity. We subsequently found that most *A. trota* isolates possessed the serine protease gene, but not the metalloprotease gene. Therefore, we determined the nucleotide sequence of the serine protease gene and its chaperone *A. trota* gene. The results obtained revealed that the deduced amino acid sequences of serine protease and the chaperone were homologous to those of *A. sobria* with identities of 83.0% and 75.8%, respectively.

## Introduction

*Aeromonas* species ubiquitously inhabit aquatic environments including fresh water, brackish water, and seawater [1], [2], [3], [4]. The mesophilic *Aeromonas* species often causes sporadic diarrhea and wound infections in both children and adults [4], [5]. *A. hydrophila* and *A. sobria* were previous shown to occasionally cause extraintestinal infections such as necrotizing soft-tissue infections and sepsis in compromised patients with diabetes and hepatic diseases, which suggested that *A. hydrophila* and *A. sobria* are the most pathogenic species [4], [5], [6], [7]. The genome of *A. hydrophila* ATCC7966 encodes various virulence factor genes including hemolysin, proteases, and lipases [8]. Previous studies demonstrated that *Aeromonas* produced various extracellular proteins containing hemolysin, protease, and lipases [9], [10]. As virulent factors, the hemolysins produced by *A. hydrophila* and *A. sobria*, aerolysin and aerolysin-like hemolysin (ALH), were shown to induce the accumulation of fluid in the intestinal loop test, and were, therefore, considered to be major etiological agents of diarrhea [11], [12]. *A. sobria* and *A. hydrophila* also produce extracellular serine protease and metalloprotease and the properties of these proteases have been studied. The serine protease produced by *A. sobria* (ASP), which is defined as a member of the kexin subfamily of serine proteases, induced edema at the site of injection when it was injected subcutaneously [13], [14]. This symptom was attributed to activation of the prekallikrein-kininogen cascade [14], [15]. In contrast, the metalloprotease produced by *A. hydrophila* (AMP) was shown to be involved in the degradation of elastin, a constitutive insoluble protein [16].

*A. trota* is a mesophilic species that is sensitive to ampicillin even though most *Aeromonas* species are resistant to ampicillin because of the production of beta-lactamase [4]. Previous studies demonstrated that *A. trota* was enterotoxic in an animal model, and was consequently considered to be the causative species of diarrhea [17], [18]. However, a correlation does not always exist between the enterotoxicity and hemolytic activity of *A. trota.* Therefore, further studies on the pathogenicity of *A. trota* are warranted in order to identify the etiological agent responsible for its diarrheagenicity. In this study, we examined the properties of the hemolysin of *A. trota* and showed that the etiological agent of *A. trota*-induced diarrhea is the hemolysin produced in the intestinal tract. We also examined the proteolytic properties of *A. trota* and determined the sequence of the *A. trota* serine protease gene.

*A. trota* was previously shown using genotypic and phenotypic analyses to be identical to *A. enteropelogenes* [19], [20]. We use the species name *A. trota* in this study because it has been cited more frequently.

## Materials and Methods

### Bacterial Strains

*A. trota* 404, 701, 702, 703, 714, 715, 716, and 717 and *A. sobria* 288 were isolated from patients with diarrhea. *A. trota* 704, 705, 706, 707, 708, 709, 710, 711, 712, and 713 and *A. hydrophila* 453 were isolated from environmental water and soil. *A. trota* ATCC49657 was obtained from the American Type Culture Collection (Manassas, VA, USA). The bacterial species of strains used in this study were identified by us using the restriction fragment length polymorphism of PCR-amplified 16S rRNA gene or using the sequencing of 16S rRNA gene [21].

These bacteria were cultured in nutrient broth medium (NB) (Eiken Chemical Co., Ltd., Tokyo, Japan) with shaking or nutrient agar medium (NA) (Eiken Chemical Co.) at 37°C.

### Hemolytic Activity of Cells on Solid Medium

The hemolytic activity of bacteria was assayed using NA containing 5% (v/v) sheep erythrocytes. Strains were cultured in NB at 37°C for 20 h and 2 µL of these cultures were dripped on blood agar plates. The hemolytic activity of bacteria was assessed after incubation at 37°C for 24 h by the appearance of a transparent zone around the bacteria.

### Proteolytic Activity of Cells on Solid Medium

The proteolytic activity of bacteria was assayed using NA containing 1% skim milk. Strains were cultured in NB at 37°C for 20 h and 2 µL of these cultures were dripped on skim milk agar plates. The proteolytic activity of bacteria was assessed after incubation at 37°C for 24 h by the appearance of a transparent zone around the bacteria.

### Preparation of Culture Supernatants and Cell Lysates

After bacteria were cultivated in liquid medium, a portion of the culture was taken at the period indicated in the text and centrifuged at 15,000×g for 5 min at 4°C. The culture supernatant was recovered, and the cell pellet was suspended in 10 mM Tris-HCl buffer (pH 7.4). The cell suspension was sonicated and centrifuged at 15, 000×g for 10 min at 4°C. The supernatant was then used as a cell lysate sample.

### Hemolytic Assay of Liquid Samples

The hemolytic activity of liquid samples was measured by the procedure described previously [12]. Aerolysin and ALH produced by *A. hydrophila* and *A. sobria*, respectively, were secreted extracellularly as inactive preforms with the carboxy-terminal (C-terminal) domain, which was then cleaved off to convert these hemolysins to active forms [22]. The hemolytic activities of samples were measured with and without the pretreatment with trypsin (2 µg/ml) at 37°C for 1 h in order to elucidate the net hemolytic activities of samples containing the preforms of hemolysin. Briefly, samples were serially diluted two-fold with the 10 mM Tris-HCl buffer (pH7.4) containing 0.9% NaCl. A total of 100 µL of each sample was mixed with an equal volume of 1% (v/v) sheep erythrocyte solution suspended in the same buffer. After incubation at 37°C for 1 h, the mixture was centrifuged at 1, 000×g for 5 min at 4°C. The supernatant was collected and its absorbance was measured at 540 nm. One hemolytic unit was defined as the amount of toxin causing 50% hemolysis under these conditions.

### Detection of Hemolysin and Serine Protease by Immunoblotting

Hemolysin was detected by immunoblotting using antiserum against the ALH produced by *A. sobria*. Antiserum against ALH, which was used in this study, was prepared in a previous study [12]. Antiserum against serine protease was prepared by injecting the conjugant of the peptide CYPDLSVRDLRDLLARSATR-NH_2_ and keyhole limpet hemocyanin into rabbits. The peptide was composed of 19 amino acid residues corresponding to the 377th to 395th residues of the amino acid sequence of *A. trota* serine protease deduced in this study. The amino acid sequence of the peptide was also conserved in the *A. sobria* serine protease sequence. Preparation of the antiserum was entrusted to Sigma-Aldrich Co. (St. Louis, MO, USA).

The proteins in the culture supernatant were precipitated by a treatment with trichloroacetic acid (TCA). The TCA solution was added to 1.0 mL of the culture supernatant to reach a concentration of 10%. The mixture was left for 30 min at room temperature and the insoluble materials yielded were collected by centrifugation. After rinsing with ethanol, the precipitates were solubilized with 100 µL loading solution for sodium dodecyl sulfate polyacrylamide gel electrophoresis (SDS- PAGE). SDS samples of cell lysates were prepared by adding the loading solution for SDS-PAGE to the cell lysates. The concentration of acrylamide in the gel was 15%. A portion of the sample was loaded onto SDS-polyacrylamide gel lanes. After electrophoresis, the proteins in the gel were transferred to a polyvinylidene fluoride (PVDF) membrane (Millipore Corporation, Billerica, MA, USA) on a trans-blot apparatus for 30 min at 160 mA at room temperature. The membranes were reacted with antiserum against ALH or the serine protease peptide and then with horseradish peroxidase-conjugated donkey anti-rabbit immunoglobulin G (IgG) (GE Healthcare, UK Limited, Buckinghamshire, UK), as described by Towbin *et al*. [23].

### Assay for Enterotoxic Activity using Bacteria

A mouse intestinal loop assay was performed as described previously [12] to elucidate the enterotoxic activity of bacteria. All experiments were approved by the Institute Animal Care and Use Committee, Tokushima Bunri University.

*A. trota* 701 was cultivated in NB at 37°C and cells were then collected. These cells were suspended at a concentration of 3×10^7^ cells/0.1 mL, and 0.1 mL of either antiserum against ALH or pre-immunization serum was added.

Mice were anesthetized with sodium pentobarbital, and the intestines were exteriorized through a midline incision. The intestinal lumen was rinsed three times with saline, after which a series of ligated intestinal segments (loops) approximately 3 cm long was created. One or two loops were created per intestine and each loop was injected with 0.2 ml of the sample solution described above. Mice were killed 3 h after the injection and the weight of each loop was measured. When the ratio of the weight of the loop (in grams) to its length (in centimeters) was more than 0.20, the injected sample was regarded as a causative agent inducing a positive response.

### Detection of the Protease Gene by Colony Hybridization

Strains containing positive and negative controls were inoculated onto NA plates and incubated at 37°C for 24 h. Colonies were transferred onto a Hybond-N^+^ nylon membrane (GE Healthcare). The membrane was then processed for cell lysis, the denaturation of DNA, and neutralization. The processed membrane was baked at 120°C for 1 h and washed for 3 h at 68°C with 3×standard saline citrate containing 0.1% SDS. The membrane with the colony blots was hybridized using 15–25 ng/ml labeled DNA probes. Hybridized probes were identified using a chemiluminescent detection method with the DIG Luminescent Detection Kit (Roche Molecular Biochemicals, Mannheim, Germany) following the manufacturer’s instructions.

DNA probes were prepared using a PCR digoxigenin probe synthesis kit (Roche Applied Science, Penzberg, Germany) according to the manufacturer’s instructions. The primers AP-165 (5′-ccctccaacagcaacttctggaacctggtg-3′) and AP-166 (5′-tccgggtaggcggacatcagcagcgccatg-3′) were used for PCR amplification of the serine protease gene and ASMP-03 (5′-aggacgccaccggcccggggggcaa-3′) and ASMP-04 (5′-gaccagccagtcgttgctcccctt-3′) were used for PCR amplification of the metalloprotease gene. The chromosomal DNA of *A. sobria* 288, which possesses both the serine protease and metalloprotease genes, was used as a template. The DNA probe for the serine protease gene covered the region from 814th to 1,136th of its nucleotide sequence (+1 nucleotide was A of the initiation codon of translation) and that for the metalloprotease gene covered the region from 557th to 1,107th of its nucleotide sequence.

### Cloning of the Serine Protease Gene in *A. trota* and Determination of its Nucleotide Sequence

The chromosomal DNA of *A. trota* 701 was prepared from cells cultivated in the NB using the DNA extraction kit ISOPLANT II (Wako Pure Chemical Industries Ltd., Osaka, Japan). The chromosomal DNA extracted was digested with *Sac*I and *Hin*dIII and the fragment generated was cloned into the pUC119 digested with the same restriction enzymes. Recombinant plasmids were transformed into *Escherichia coli* HB101 by the electroporation method. Transformants containing the plasmid harboring the serine protease gene were selected by colony hybridization as described above. The plasmid harboring the serine protease gene was isolated from its transformant using the QIAGEN plasmid kit (Qiagen, Venlo, Netherlands) according to the manufacturer’s protocol. The nucleotide sequence of the DNA fragment containing the serine protease gene was subsequently determined by the dideoxy chain termination method using BigDye v1.1 (Applied Biosystems, Forester, CA, USA).

## Results

### Hemolytic and Proteolytic Activities of *A. trota* on Solid Medium

**Figure 1:**
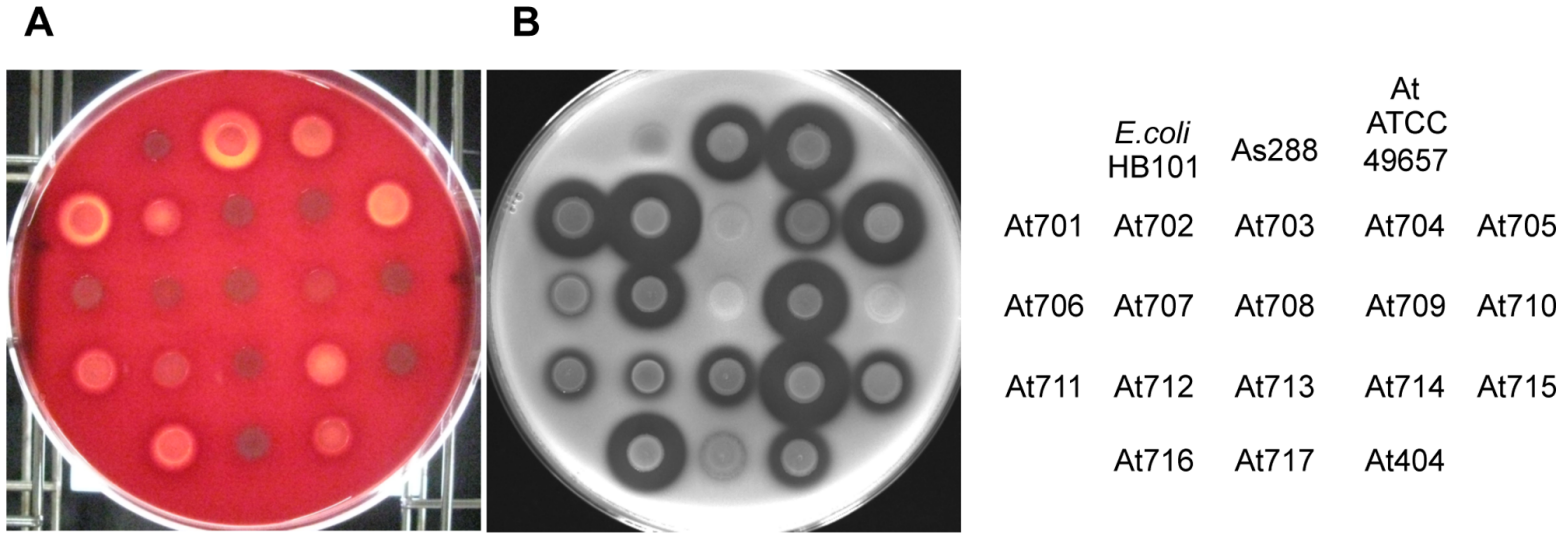
Cultivation of *A. trota* on agar medium containing erythrocytes or skim milk. Each strain was cultivated in nutrient broth for 20 µL of culture solutions were dropped on each agar medium. After inoculation, these plates were incubated at 37°C for 24 h. Hemolytic activity (A) and proteolytic activity (B) were assessed by the appearance of a transparent zone around the bacteria on each plate, respectively. The table at the right side of the figure shows the names of the strains used.

To examine the hemolytic and proteolytic activities of *A. trota*, 19 strains of *A. trota* were cultivated at 37°C on solid medium containing erythrocytes or skim milk, respectively. *A. sobria* 288, which produces hemolysin, serine protease, and metalloprotease, was used as a positive control strain and *E. coli* HB101 was as a negative control strain. As shown in Figure 1(A), *A*. *sobria* 288 presented a hemolytic zone around the bacteria on medium containing erythrocytes and a transparent zone on medium containing skim milk, whereas *E. coli* HB101 did not. Among the *A. trota* strains tested, nine strains (strains ATCC49657, 701, 702, 705, 711, 712, 714, 716, and 404) created hemolytic zones while the others did not. The hemolytic zone formed by *A. trota* was narrower than that formed by *A. sobria* 288, which indicated that the hemolytic activity of *A. trota* was weak. On the other hand, 15 of 19 *A. trota* (strains ATCC49657, 701, 702, 704, 705, 706, 707, 709, 711, 712, 713, 714, 715, 716, and 404) formed transparent zones around the bacteria on solid medium containing skim milk (Figure 1(B)). The sizes of transparent zones varied and the sizes of the transparent zones formed by several *A. trota* strains were similar to that formed by *A. sobria* 288.

### Hemolytic Activity of the Culture Supernatant of *A. trota* and Detection of Hemolysin by Immunoblotting

**Figure 2:**
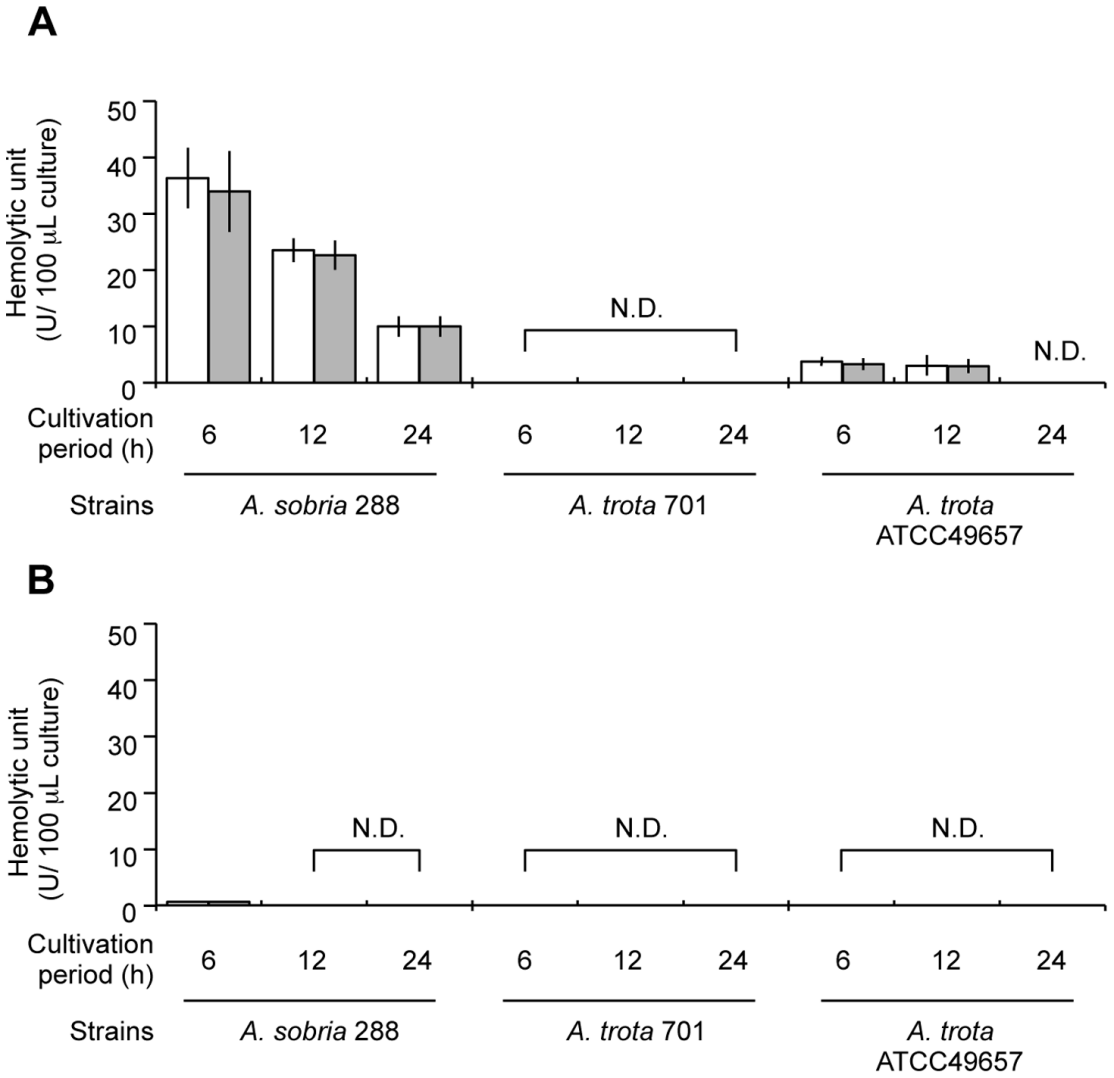
Hemolytic activity of the culture supernatants and cell lysates of *A. sobria* and *A. trota*. *A. sobria* 288, *A. trota* 701, and *A. trota* ATCC49657 were cultivated in NB medium at 37°C. A portion of the culture was collected at the period indicated and the culture supernatant (A) and cell lysate (B) were prepared as described in the text. Samples were divided into two tubes; one was incubated with trypsin at 37°C for 1 h (gray bar) and the other was kept on ice (white bar). Each sample was diluted with 10 mM Tris-HCl (pH7.4) containing 0.9% NaCl, and the hemolytic activities of serially twice diluted samples were examined, as described in the text. The hemolytic activity of a sample equivalent to 100 µL culture was calculated. N.D. shows the samples in which activity was not detected. Experiments were carried out in triplicate independently, and data were represented as an arithmetic mean ± standard deviation.

**Figure 3:**
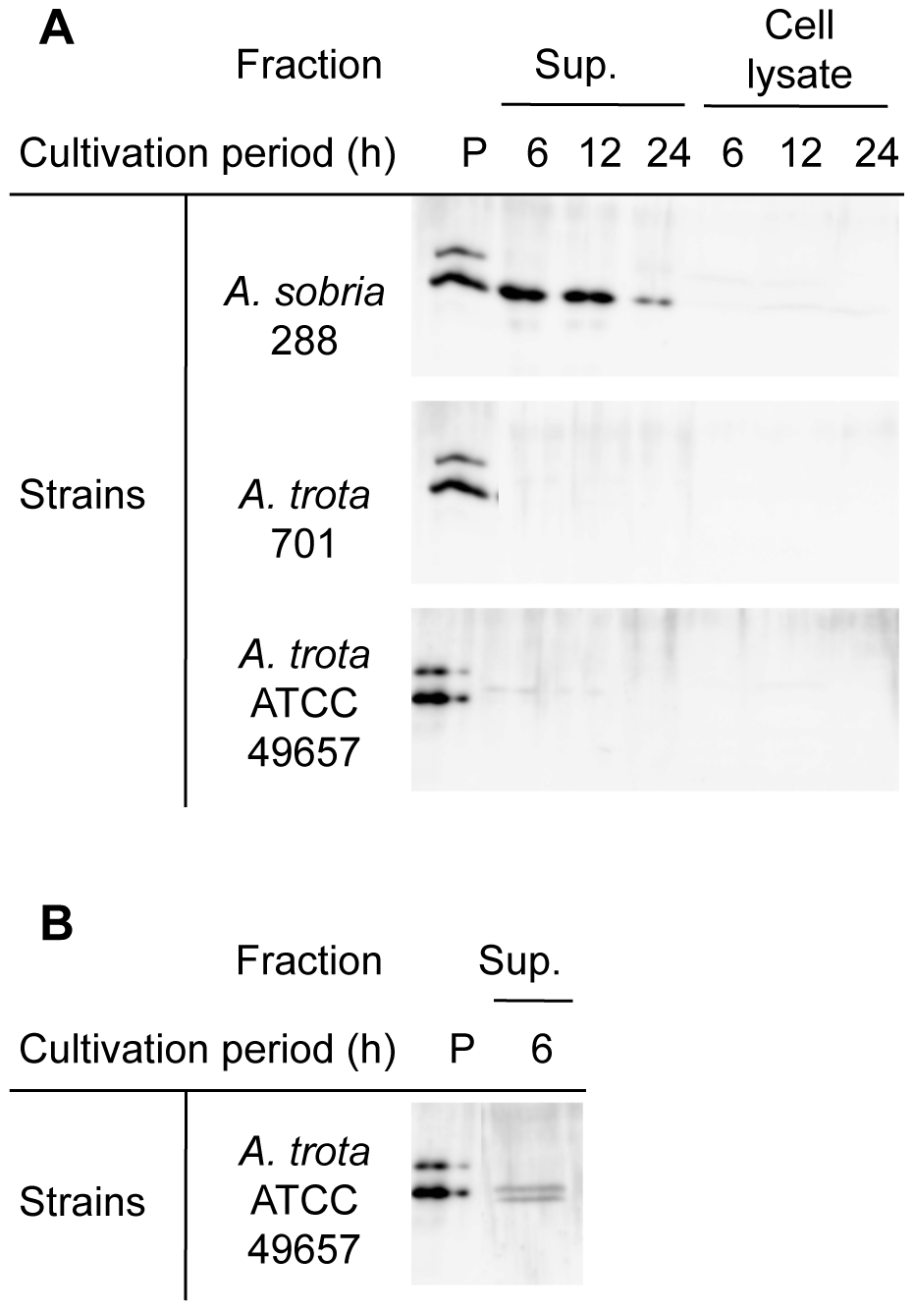
Immunodetection of hemolysins produced by *A. sobria* and *A. trota*. *A. sobria* 288, *A. trota* 701, and *A. trota* ATCC49657 were cultivated in NB medium at 37°C. A portion of the culture was collected at the period indicated and the culture supernatant and cell lysate were prepared as described in the text. Each SDS sample, which was equivalent to 50 µL culture, was applied to the lane (A). After SDS-PAGE, hemolysin was detected in each lane using anti-ALH antiserum, as described in the text. Larger amounts of SDS samples, equivalent to 250 µL culture, were applied to the lanes of SDS-PAGE for clearer detection (B). The sample containing pre-ALH and ALH was applied to lane P as a positive control. The upper band in lane P is pre-ALH and lower band is mature ALH.

*A. trota* 701, *A. trota* ATCC49657 and *A. sobria* 288 were cultivated in liquid medium at 37°C for the time indicated in Figure 2. Supernatants and cell lysates of these cultures were prepared and the hemolytic activities of these samples were measured. As shown in Figure 2, the culture supernatant of *A. sobria* 288 showed strong hemolytic activity, whereas that of the cell lysate was weak. The hemolytic activity of the culture supernatant of *A. sobria* 288 was high at the early period (6 h), and thereafter decreased in a time-dependent manner. Although *A. trota* 701 and *A. trota* ATCC49657 showed hemolytic activity in the culture on solid medium containing erythrocytes (Figure 1(A)), their activities were markedly lower in the liquid culture. The culture supernatant of *A. trota* ATCC49657 showed slight hemolytic activity whereas the cell lysate of the strain did not show any activity. Hemolytic activity was not observed in *A. trota* 701 samples (Figure 2). We subsequently detected hemolysin in these culture supernatants and cell lysates by immunoblotting using anti-ALH antiserum. As shown in Figure 3(A), the active form of ALH was detected in the culture supernatant of *A. sobria* 288, which demonstrated that the detection of hemolysin was compatible with hemolytic activity. A faint band was detected in the culture supernatant of *A. trota* ATCC49657 after a 6-h cultivation, which indicated that *A. trota* produced homologous hemolysin extracellularly, and the hemolysin produced by *A. trota* ATCC49657 reacted with anti-ALH antiserum (Figure 3(B)).

### Sequencing Analysis of the Hemolysin Gene in *A. trota* 701 and *A. trota* ATCC49657

A. A. Khan *et al*. previously reported the nucleotide sequence of the hemolysin gene of *A. trota* (GenBank accession number AF064068), and showed that it was homologous to the *alh* gene of *A. sobria* [24]. Therefore, we designed PCR primers according to its sequence and amplified DNA fragments from *A. trota* 701 and *A. trota* ATCC49657. The nucleotide sequences of the amplified DNA fragments were determined. The nucleotide sequences (1,476 bp) of *A. trota* 701 and *A. trota* ATCC49657 were 99.1% and 99.0% identical to the gene of *A. trota* hemolysin previously reported, and 67.3% and 67.6% identical to the *alh* gene of *A. sobria* (GenBank accession number AAN77507), respectively. At the level of the deduced amino acid sequence, the hemolysins produced by *A. trota* 701 and *A. trota* ATCC49657 showed 100% and 99.4% identities to that produced by *A. trota*, and 61.9% and 61.9% f identities and 92.7% and 93.1% similarities to that produced by *A. sobria*, respectively.

### The Enterotoxicity of *A. trota* 701 and Inhibitory Effect of Anti-ALH Serum

**Figure 4:**
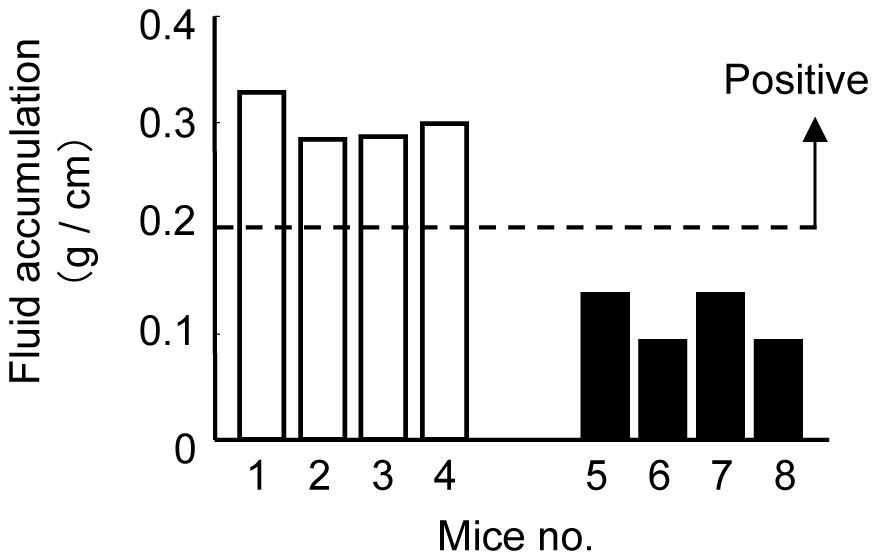
Enterotoxic activity of *A. trota* 701. Enterotoxic activity was elucidated using the mouse intestinal loop test. A total of 3×10^7^ cells of *A. trota* 701 was mixed with either pre-immunization serum (white bar) or anti-ALH serum (black bar), and ingested into the mice intestinal loop. When the accumulation of fluid induced by the sample was more than 0.2 g/cm, the sample was considered to be enterotoxigenic. One group consisted of four mice.

The enterotoxicity of *A. trota* 701 was examined using the mouse intestinal loop test. When the accumulation of fluid induced by the sample was more than 0.2 g/cm, the enterotoxic activity of the sample was considered to be positive. As shown in Figure 4, an injection of living *A. trota* 701 with pre-immunized serum induced positive fluid accumulation in all mice tested, which indicated that *A. trota* 701 is a diarrheagenic strain. Fluid accumulation was not observed in the samples when *A. trota* 701 was injected with anti-ALH antiserum. Anti-ALH antiserum did not affect the bacterial growth of *A. trota* (data not shown). This result confirmed that the hemolysin produced by *A. trota* 701 in the intestinal tract plays an essential role in the diarrhea induced by living cells.

### Detection of the Serine Protease Gene of *A. trota* and Determination of its Nucleotide Sequence

As shown in Figure 1(B), most *A. trota* isolates produced a transparent zone on the solid medium containing skim milk. Moreover, as shown in Figure 3(B), the culture supernatant of *A. trota* ATCC49657 contained the active form of hemolysin, which is the cleavage product of the precursor. These results suggested that protease was secreted outside of the cell. Therefore, we investigated the protease produced by *A. trota*.

**Figure 5:**
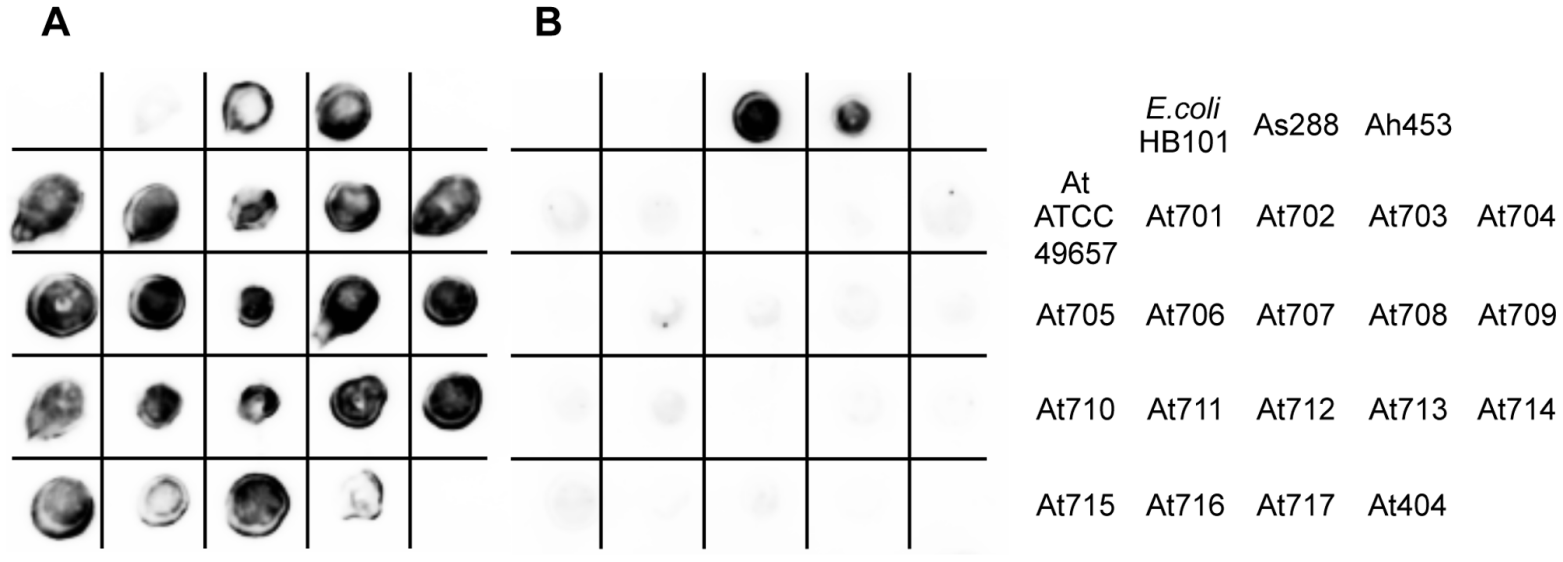
Detection of the protease genes of *A. trota* by colony hybridization using specific probes. Each strain was inoculated on the NA plate and incubated at 37°C for 24 h. Bacterial colonies were transferred onto the Hybond-N^+^ membrane, and bacterial DNAs were baked. The colony reacted with specific probes for either the serine protease gene (A) or metalloprotease gene (B), as described in the text. The table on the right side of the figure shows the names of the strains used.

Serine protease and metalloprotease are the main extracellular proteases of *A. sobria*, [25]. We investigated whether the genomic DNA of *A. trota* reacted with specific probes prepared from the serine protease and metalloprotease genes of *A. sobria*. *A. sobria* 288 and *A. hydrophila* 453, which are known to possess both serine protease and metalloprotease genes, were used as positive control strains and *E. coli* HB101 was used as a negative control strain. As shown in Figure 5(A), 17 of 19 *A. trota* reacted strongly with the probe for the serine protease gene, whereas the remaining 2 only reacted weakly. None of the *A. trota* isolates tested reacted with the probe for the metalloprotease gene, while a reaction was observed between this probe and the genomic DNA from *A. sobria* and *A. hydrophila* (Figure 5(B)).

**Figure 6:**
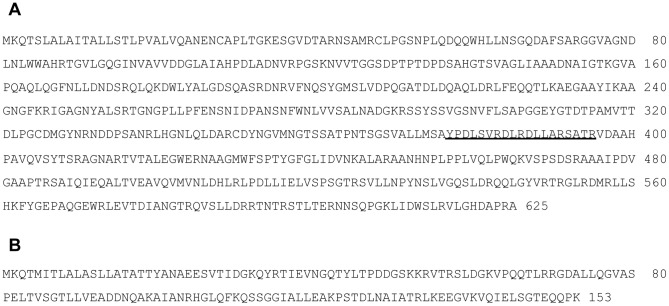
Deduced amino acid sequence of serine protease and *A. trota* chaperone. The sequence (A) is the deduced amino acid sequence of serine protease and the sequence (B) is that of *A. trota* chaperone. Amino acid sequences are numbered as 1 from the initiator Met, and the numbers on the right side of the amino acid sequences indicate the number of amino acid residues from A of the initiation Met. The nucleotide sequence encoding these genes was deposited in GenBank (Accession No. KF914659). Underlined sections represent the amino acid sequence used to make anti-serine protease peptide antiserum.

We subsequently cloned the gene encoding serine protease using the shotgun cloning method, as described in the Materials and Methods, and determined the nucleotide sequence of the gene. In the case of *A. sobria*, the chaperone gene, which is involved in assisting ASP to construct the active form, is known to be encoded in the 3′ flanking region of the gene encoding serine protease [26]. Similar to *A. sobria*, the nucleotide sequence of *A. trota* determined in this study showed that the open reading frame, which is homologous to the chaperone gene of *A. sobria,* was located in the same region. The nucleotide sequence from the start codon of the serine protease gene to the stop codon of the chaperone gene (2,358 bp) of *A. trota* 701, which was deposited in GenBank (Accession No. KF914659), showed 80.2% identity to that of *A. sobria* (GenBank accession number AF253471). The deduced amino acid sequences of serine protease and its chaperone were shown in Figure 6. The deduced amino acid sequences of serine protease and its *A. trota* chaperone had 83.4% and 76.5% identities to those of *A. sobria*, and 97.8% and 92.8% similarities, respectively.

**Figure 7:**
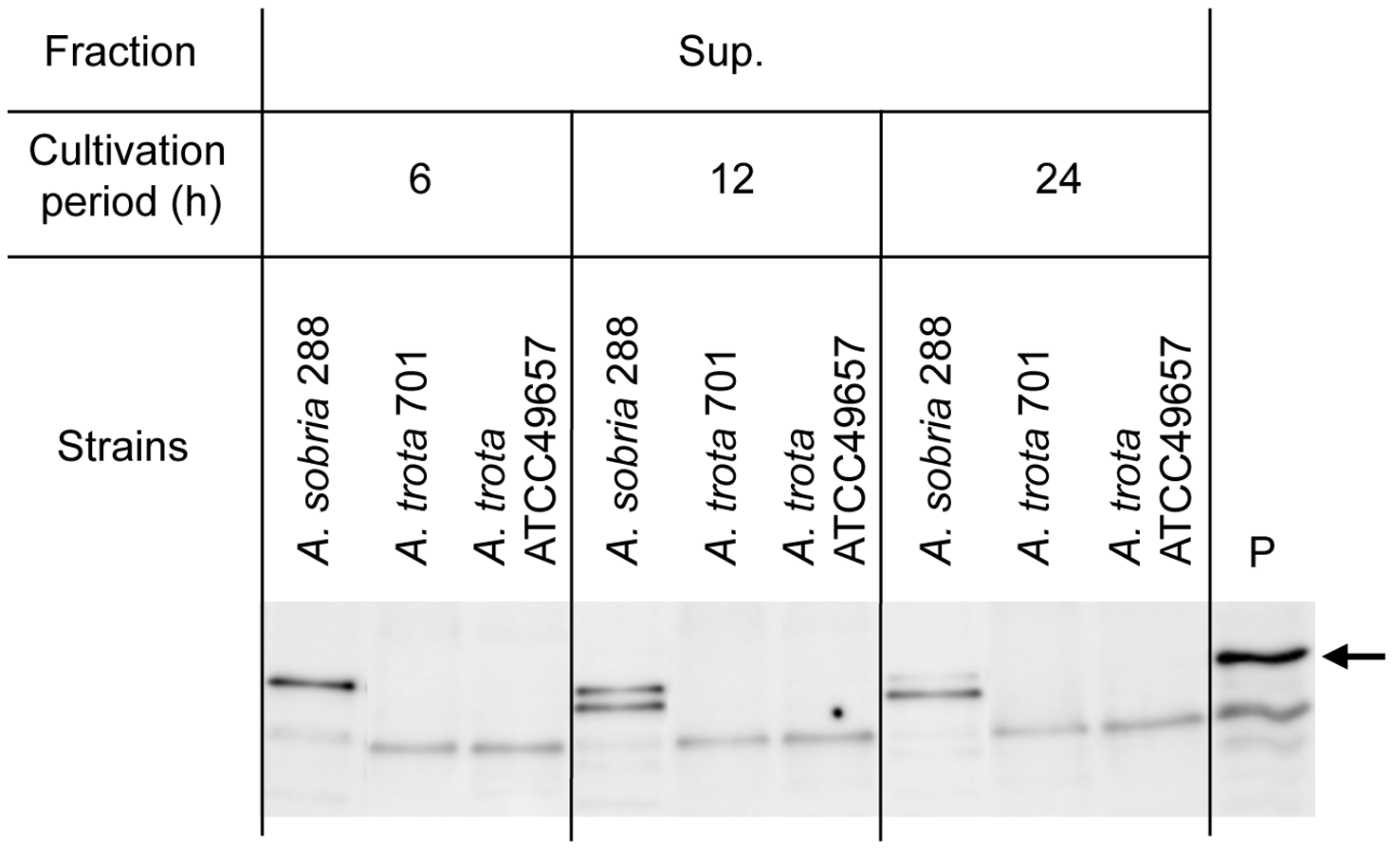
Immunodetection of serine protease in the culture supernatants of *A. sobria* and *A. trota*. *A. sobria* 288, *A. trota* 701, and *A. trota* ATCC49657 were cultivated in NB medium at 37°C. A portion of the culture was collected at the period indicated and the culture supernatant was prepared as described in the text. Each SDS sample, which was equivalent to 50 µL culture, was applied to the lane. After SDS-PAGE, serine protease was detected in each lane using anti-serine protease peptide antiserum, as described in the text. The sample containing *A. sobria* serine protease was applied to lane P as a positive control. The band along with the arrow indicated the *A. sobria* serine protease with a molecular size of 64 kDa.

To elucidate the extracellular production of the *A. trota* serine protease, we detected serine protease in the culture supernatant by immunoblotting using antiserum against the serine protease peptide, as described in Figure 6(A). When *A. sobria* 288 was cultivated in liquid medium at 37°C, the culture supernatant obtained showed a serine protease band with a molecular mass of 64 kDa, as shown in Figure 7. The band with a molecular mass of 64 kDa disappeared with the longer cultivation, and the band with a smaller molecular mass appeared. The disappearance of the 64 kDa serine protease and appearance of a smaller molecule did not occur in the *A. sobria* 288 metalloprotease-deficient mutant, even after the longer cultivation, which suggests that the serine protease was degraded by the metalloprotease (data not shown). On the other hand, when *A. trota* 701 and *A. trota* ATCC49657 were cultivated in liquid medium at 37°C, the culture supernatant obtained did not show a serine protease band with a molecular mass of 64 kDa by immunoblotting using anti-serine protease peptide antiserum (Figure 7); however, the band with a smaller molecular mass was detected. Consistent with the result obtained for the detection of serine protease, the culture supernatants of *A. trota* did not show any proteolytic activity against azocasein (data not shown). These results suggest that the serine protease released from *A. trota* into the culture supernatant was degraded and did not express proteolytic activity. However, the protease released in the cultivation on solid medium immediately acted on casein before being attacked by other proteases, and created a transparent zone around the colony.

As described, intact serine protease remained in the *A. sobria* 288 metalloprotease-deficient mutant strain without being attacked by proteases. Therefore, we consider the serine protease of *A. sobria* to be more stable than that of *A. trota*.

## Discussion

*A. trota* is a diarrheagenic *Aeromonas* species. However, pathological analysis of *A. trota* has not been extensive. *A. trota* was previously reported to be isolated from diarrhea patients and exhibited enterotoxicity in an *in vivo* animal model [17], [18]. The hemolysins produced by *A. sobria* and *A. hydrophila* were shown to play a role in diarrhea [11], [12]. D. V. Singh and S. C. Sanyal previously reported the absence of a correlation between the enterotoxicity and hemolytic activity of *A. trota*; therefore, the causative agent by which *A. trota* induces the accumulation of fluid remains unclear [17].

When *A. trota* was cultivated on solid medium containing erythrocytes at 37°C, 9 of 19 *A. trota* isolates lysed the surrounding erythrocytes and formed a hemolytic zone. However, most samples prepared from the culture of *A. trota* did not show hemolytic activity when cultivated in liquid medium at 37°C. Moreover, hemolysin was not detected by immunoblotting when a normal volume of sample was used (Figure 3(A)). These results indicate that the phenotype of *A. trota* in liquid medium differs from that on solid medium. On the other hand, when *A. trota* 701, which showed hemolytic activity on solid medium and non-hemolytic activity in the liquid medium, was injected into a ligated mouse intestinal tract, it induced the accumulation of fluid, and the diarrheagenic ability of *A. trota* 701 was suppressed by anti-ALH antiserum. These results confirmed that *A. trota* produced an adequate amount of hemolysin to cause the accumulation of fluid, and its hemolysin plays an important role in the enterotoxicity of *A. trota*. D. V. Singh and S. C. Sanyal reported that *A. trota,* which was not enterotoxic in the initial rabbit ileal loop test, became enterotoxic after a sequential passage through the rabbit ileal loop; however, the mechanism underlying this phenomenon is not yet clearly understood [17]. We speculate that the production of hemolysin by *A. trota* is regulated strictly, and the conditions present in the intestinal tract, which is the infectious site of *A. trota* in humans, are suitable for *A. trota* to produce hemolysin.

We failed to purify the hemolysin produced by *A. trota* in the present study because *A. trota* did not produce a sufficient amount of hemolysin in liquid medium. Further studies using purified hemolysin are warranted to elucidate the enterotoxicity of *A. trota* in more detail.

We also examined the properties of the extracellular protease produced by *A. trota*. When *A. trota* was cultivated on solid medium containing skim milk at 37°C, 15 of 19 *A. trota* isolates digested the skim milk around bacteria and formed a transparent zone. We subsequently demonstrated that most *A. trota* strains possessed the gene encoding serine protease, but not that for metalloprotease, and then determined the nucleotide sequence of the *A. trota* serine protease gene. However, we could not detect serine protease by immunoblotting in both the culture supernatant and cell lysate prepared from the *A. trota* culture at 37°C. V. Husslein *et al.* previously reported the purification of serine protease from the culture supernatant of *A. trota,* which had a molecular mass of 70 kDa [27]. However, the conditions used for the *A. trota* culture were not described. *A. trota* may sense preferable conditions to produce serine protease. Previous studies demonstrated that the serine protease produced by *A. sobria*, which we previously termed ASP, cleaved several plasma proteins and activated several intrinsic cascades containing the kallikrein-kinin cascade [14], [15], [28], [29], [30]. This was shown to induce physiological reactions such as histamine release and the induction of edema in the rat dermis [14], [15]. Wound infection and septic shock have been reported in *A. trota* infections [31]. It is likely that *A. trota* produces serine protease in infectious sites, and this may subsequently contribute to the pathogenicity of *A. trota* via its activity to cleave physical proteins, such as ASP.

Although hemolysin and serine protease were not detected in the culture supernatant of *A. trota*, it induced hemolysis and proteolysis on the solid medium and exhibited enterotoxic activity via the ability of hemolysin *in vivo*. The reason why hemolysin and serine protease were not detected in the culture supernatant of *A. trota* remains unknown; however, homologous proteins were detected in the *A. sobria* culture. The inability to detect hemolysin and serine protease in *A. trota* may be related to the stability against other proteases and the amount produced extracellularly. Future studies are needed to clarify these problems.
